# Occurrence and Molecular Variability of the Main Kiwifruit Viruses in the Sichuan Province of China

**DOI:** 10.3390/v14112460

**Published:** 2022-11-06

**Authors:** Jing Shang, Qi Jia, Lei Zhang, Siqi Zhang, Junbo Du, Wenming Wang, Jing Shui

**Affiliations:** 1 College of Agronomy, Sichuan Agricultural University, Chengdu 611130, China; 2 State Key Laboratory of Crop Gene Exploration and Utilization in Southwest, Chengdu 611130, China; 3 Bureau of Agriculture and Rural Affairs, Luzhou 646000, China

**Keywords:** kiwifruit virus, genetic variation, structural prediction

## Abstract

Viruses cause important yield losses in kiwifruit. Here, we studied the occurrence and population structure of the major kiwifruit viruses in the Sichuan province of China. RT-PCR results showed the presence of Actinidia virus A (AcVA), Actinidia virus B (AcVB), Actinidia chlorotic ringspot-associated virus (AcCRaV), and the cucumber mosaic virus (CMV). AcCRaV was widely distributed, followed by CMV. These two viruses were often detected in co-infection with AcVA and AcVB. The virus detection rate was positively correlated with vine age. Four phylogenetic groups of AcVA and AcVB were identified, with AcVA isolates clustering mainly in subgroup I, and AcVB isolates clustering mainly in subgroups II, III, and IV. All CMV isolates clustered in subgroup II, and AcCRaV isolates clustered in subgroup IA. The genome of AcVA and AcCRaV was under negative selection pressure, while the genome of AcVB and CMV was under positive selection pressure. All the viruses, except AcVB, were in a state of expansion. The full-length genome of the most widely distributed AcCRaV isolate in kiwifruits in the Sichuan province was characterized by sequencing. Unique eight-nucleotide (TTTTTGAT) repeats were found in the 5′-terminal non-coding region of the AcCRaV RNA3 in a possible association with reduced disease symptoms. This is the first study of kiwifruit viruses in Sichuan.

## 1. Introduction

The Sichuan and Shaanxi provinces produce about 75% of China’s kiwifruit. Kiwifruit is highly susceptible to infection by fungi, bacteria, and viruses [1]. Most previous studies have focused on fungal diseases and bacterial ulcers of kiwifruit. The population structure and incidence of kiwifruit viruses are still unclear. Kiwifruit virus diseases can cause fruit malformation, resulting in reduced yield and quality. Both seeds and propagation materials can carry viruses, increasing the risk of transmission and epidemics of viral diseases [2]. The kiwifruit variety “Hongyang” has good agronomic characteristics, but poor disease resistance. In recent years, the large-scale planting of this variety in the Sichuan province has led to an increase in the incidence of kiwifruit virus diseases.

Viruses that specifically infect kiwifruit are mostly from the *Betaflexiviridae* family, such as Actinidia virus A (AcVA), Actinidia virus B (AcVB), and the Actinidia seedborne latent virus (ASbLV) [3,4,5]. Infected kiwifruits usually show bright leaf veins, chlorotic ringspots, and spots [6]. The citrus leaf blotch virus (CLBV) of the kiwifruit genus causes symptoms of pulsing, mosaic, and interveinal chlorosis [7,8,9]. Actinidia chlorotic ringspot-associated virus (AcCRaV) usually causes chlorosis and ringspot symptoms [10]. Later, it was found that Actinidia Emaravirus 2 (ACEV-2) could infect kiwifruit and promote the emergence of new viruses due to frequent gene recombination [11,12,13]. Actinidia virus 1 (ACV-1) was also recently reported as a novel kiwifruit virus [14].

Other kiwifruit viruses include cucumber mosaic virus (CMV) [15], apple stem grooving virus (ASGV) [16,17], potato virus X (PVX) [18], cucumber necrosis virus (CNV), alfalfa mosaic virus (AMV) [19,20,21], and citrus tatter leaf virus (CTLV), among many others [22,23,24,25,26].

The genetic diversity of virus populations is affected by many factors, including selection pressure and gene flow [27,28,29,30,31,32]. Studying the genetic diversity and molecular variation of virus populations is helpful for understanding the population dynamics and evolutionary mechanism of viruses [33]. The main viruses infecting kiwifruit, their occurrence, and population structure in the Sichuan province have not been studied. Here, we investigated the kiwifruit viruses in the main kiwifruit-producing areas in the Sichuan province (Pujiang, Qionglai, Cangxi, Dujiangyan, and Yingjing). Their occurrence and genome variability were also studied.

## 2. Materials and Methods

### 2.1. Sample Collection

Kiwifruit leaf samples with vesicular shrinkage, ringspots, leaf roll, chlorotic spots, narrow and long deformities, vein yellowing, necrosis, and other symptoms were collected from Dujiangyan City, Qionglai City, Ya’an City, Cangxi County, and Pujiang County in the Sichuan province, China. Leaf samples were stored in dry bottles with anhydrous calcium chloride, transported in an ice box at 4 °C, and preserved in a freezer at −80 °C.

### 2.2. Detection of Viruses by Reverse Transcriptase Polymerase Chain Reaction (RT-PCR)

Total RNA was extracted from leaves using a previously described method [34]; 4 μg of total RNA was used as a template. The primer, 5X All-in-One RT MasterMix (ABM G490, Canada) and 4 μL of ddH_2_O were added to bring the reaction system up to 10 μL for cDNA synthesis. PCR was performed as follows: 94 °C for 3 min for denaturation, followed by 35 cycles of 94 °C for 30 s, 50 °C for 30 s, and 72 °C for 1 min. The CP sequences of the virus were compared by DNAMAN software. Primers were designed using Premier 5.0 software according to conserved virus genome regions. The primers were synthesized by Shenggong Bioengineering Co., Ltd. (Shanghai, China). The primer sequences used for amplifying the viruses are listed in Table S1, including primers for CMV, AcVA, AcVB, AcCRaV, ASGV, AVX, CTLV, AMV, and CNV.

### 2.3. Cloning and Sequencing

The PCR products were analyzed by gel electrophoresis, purified by a DNA gel extraction kit (Bio Tech Corporation, Beijing, China) and sequenced by TSINGKE Biological Technology Company (Beijing, China). For each sequence, to ensure accuracy, additional PCR and sequencing experiments were performed at least three times.

Purified PCR products of four viruses (AcVA, AcVB, AcCRaV, and CMV) were cloned and analyzed to study the molecular variability of the viruses. Samples that were RT-PCR-positive for AcVA, AcVB, AcCRaV, and CMV were selected and used for amplification of the complete coat protein (CP) gene using the CP gene primers (Table S1) and the PCR programs described above. All the amplified CP genes were cloned into the PMD19-T vector (TaKaRa Bio). The recombinant vectors were transformed into *Escherichia coli* strain JM109 and sequenced by TSINGKE Biological Technology Company (Beijing, China). For each CP gene, three clones were sequenced in both orientations to confirm the consensus sequence, then all sequences were deposited in the GenBank database. The accession numbers of the virus sequences and information on the sampling sites are listed in Tables S2–S5.

### 2.4. Calculation of the Mosaic Area and Disease Severity Classification

The classification of disease severity referred to the 9-grade classification standard. The kiwifruit virus disease classification standards are: 0, asymptomatic; Grade 1, an area of chlorosis less than 10% of the total leaf area; Grade 3, an area of chlorosis 11–25% of the total leaf area, and the leaves are slightly atrophic; Grade 5, an area of chlorosis 26–40% of the total leaf area; Grade 7: an area of chlorosis 41–65% of the total leaf area, and leaf atrophy is more serious; Grade 9, an area of chlorosis over 65% of the total leaf area, leaves are curled and/or necrotic. The chlorotic areas on the collected kiwifruit leaves were selected using Photoshop software to take the average of the total area and the mosaic area.

$$\mathrm{The}\;\mathrm{disease}\;\mathrm{index}=\frac{\sum^{\;}\left(\mathrm{corresponding}\;\mathrm{level}\;\mathrm{number}\;\mathrm{of}\;\mathrm{infected}\;\mathrm{plants}\;\mathrm{at}\;\mathrm{each}\;\mathrm{corresponding}\;\mathrm{level}\right)}{\mathrm{total}\;\mathrm{number}\;\mathrm{of}\;\mathrm{plants}\;\times \;\mathrm{highest}\;\mathrm{level}\;\mathrm{value}}\times 100\%$$

### 2.5. Recombination and Phylogenetic Analysis

We analyzed the CP gene fragment of 96 virus samples from different vines. There were 22 samples of AcVA, 28 samples of AcCRaV, 16 samples of AcVB, and 30 samples of CMV (Tables S2–S5). The sequences of the *CP* genes were aligned using MUSCLE, as implemented in the MEGA 5.0 software. Six methods (RDP, GENECONV, MaxChi, Chimaera, SiScan, and 3Seq) were performed using the RDP4 software package to detect possible recombination events in the *CP* genes of the virus populations. Predictions were considered reliable when the results obtained by four or more of the six methods were supported by *p*-values of <10^−6^. Phylogenetic trees of the *CP* genes of the viruses were reconstructed using the neighbor-joining method with the Kimura two-parameter model implemented in the MEGA 5.0 software. To test the phylogeny, 1000 bootstrap replications were performed in total. Branches with bootstrap values less than 50% were removed [33].

### 2.6. Genetic Diversity, Genetic Differentiation of the Populations, and Gene Flow

Haplotype diversity (Hd) and nucleotide diversity (π) were used to estimate the genetic diversity [33]. The extent of genetic differentiation among populations was evaluated by FST, and the significance was examined by two permutation-based statistical tests, KST and Snn. Nm, the number of migrants successfully entering a population per generation, was used to measure the gene flow between populations. All of these values were calculated in DnaSP 5.0.

### 2.7. Analysis of the Selection Pressure and Population Demographics

To explore variations in the evolutionary constraints in the CP genes of the viruses, the ratio of the non-synonymous substitution rate (dN) to the synonymous substitution rate (dS) was calculated in DnaSP 5.0. Tajima’s D, and Fu and Li’s D and F tests, implemented in DnaSP 5.0, were used to explore the demographic history of the virus populations [33]. Tajima’s D test identified evolutionary events, such as population expansion, bottlenecks, and selection by comparing the estimated number of segregating sites with the mean pairwise difference among sequences. Fu and Li’s D and F tests are sensitive to a population’s demographic expansion and usually display a negative value in expanding populations.

### 2.8. Comparison of the Whole AcCRaV Genome

We adopted MEGA 5.0 for a genome-wide comparison of AcCRaV, using the website viewer ORFfinder of the NCBI (https://www.ncbi.nlm.nih.gov/orffinder/ accessed on 12 September 2022) for analyzing the sequence alignments.

### 2.9. Statistical Analysis

The one-way ANOVA model was used for analyses of the error in IBM SPSS Statistic 27, and the average value was taken. The significance was judged by the new complex range method (Duncan’s method) at *p* < 0.01.

## 3. Results

### 3.1. Virus Detection and Disease Index

Among the single infections, AcCRaV had the highest detection rate (41.94%), followed by CMV. The percentages of AcVA + AcVB, AcVA + AcCRaV, and AcCRaV + CMV were 5.16%, 7.10%, and 8.39%, respectively (Table 1). AcCRaV and CMV are the main kiwifruit viruses in the Sichuan province and can easily co-infect kiwifruit vines alongside other viruses. The disease index of AcCRaV and CMV was 7.92 and 5.47%, respectively.

### 3.2. Symptoms of Kiwifruit Viruses

**Figure 1:**
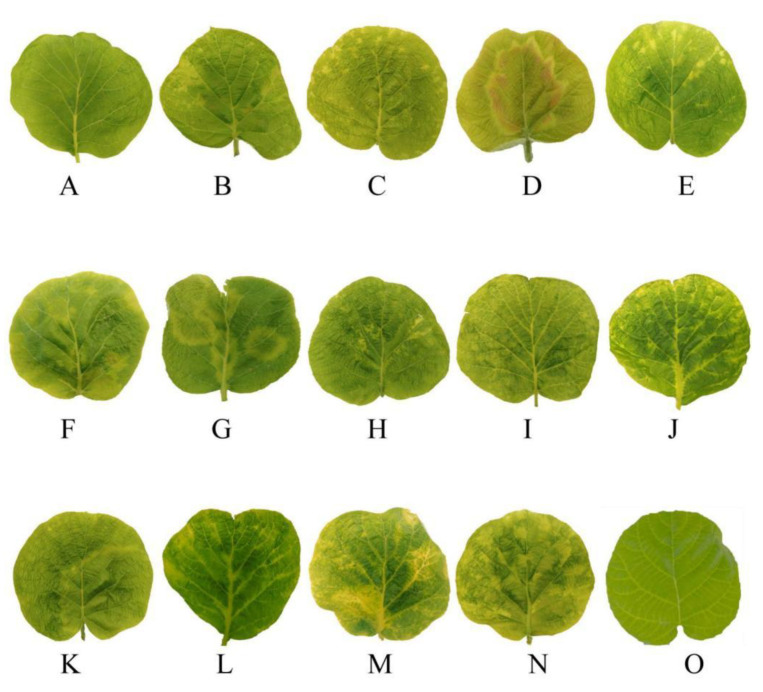
Symptoms of single and co-infections of Hongyang kiwifruits in the Sichuan province with AcVA, AcVB, AcCRaV, and CMV. Note: (**A**), AcVA; (**B**), AcVB; (**C**), AcCRaV; (**D**), CMV; (**E**), AcCRaV + AcVA; (**F**), AcCRaV + CMV; (**G**), AcVA + CMV; (**H**), AcVB + AcCRaV; (**I**), AcVB + CMV; (**J**), AcVA + AcVB; (**K**), AcVA + AcCRaV + CMV; (**L**), AcVA + AcVB + AcCRaV; (**M**), AcVB + AcCRaV + CMV; (**N**), AcVA + AcVB + AcCRaV + CMV; (**O**), normal leaves.

Four kiwifruit viruses were tested. Leaves infected with AcVA showed mild chlorosis and mild atrophy (Figure 1A). Leaves infected with AcVB showed chlorosis of the veins (Figure 1B). Leaves infected with AcCRaV showed irregular marginal spots of chlorosis (Figure 1C). The leaves infected with CMV showed large chlorotic spots and mild atrophy, and the lesion site was red (Figure 1D). Kiwifruit leaf samples infected by AcVA and AcCRaV showed slight shrinkage similar to the symptoms of AcVA, and yellow spots with irregular edges similar to the symptoms of AcCRaV (Figure 1E).

Leaf samples infected by AcCRaV and CMV showed localized local deformities and large chlorotic spots with irregular edges. With the appearance of red patches, this was similar to the symptoms of CMV (Figure 1F). The symptoms of co-infection with AcVA and CMV were some large chlorotic ringspots, and the leaves had obvious deformities (Figure 1G). Co-infection with AcVB and AcCRaV produced symptoms of chlorosis along the veins, accompanied by minor malformations (Figure 1H). The leaves showed chlorotic mottling and slight shrinkage caused by co-infection with AcVB and CMV (Figure 1I). Co-infection with AcVA and AcVB led to shrinkage and chlorosis along the veins (Figure 1J). Co-infection with AcVA, AcCRaV, and CMV showed large, chlorotic spots with wavy edges and some small chlorotic spots similar to infection with AcCRaV alone (Figure 1K). Healthy leaves were evenly smooth (Figure 1O).

### 3.3. The Detection Rate Was Affected by Vine Age and Altitude

**Figure 2:**
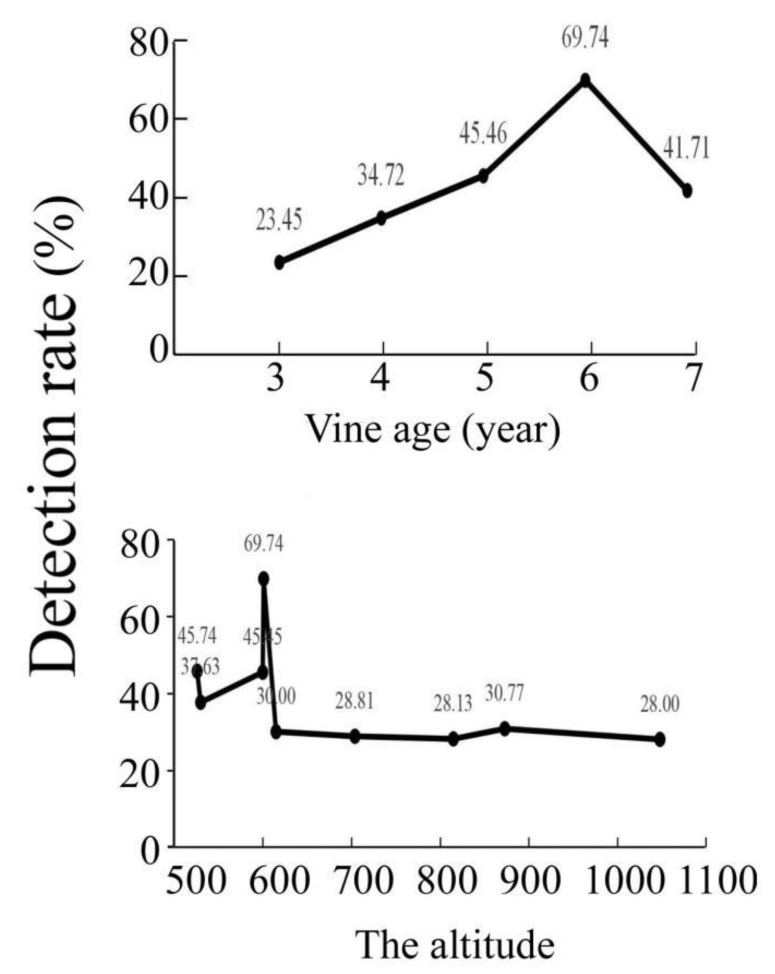
The virus detection rate was affected by the altitude and the vines’ age.

At the same altitude (600 m), the virus detection rate was related to the age of the kiwifruit vines (Figure 2). As the altitude increased, the temperature decreased. Low temperatures inhibited the activity of vectors, such as aphids and leafhoppers [35]. This may be associated with a decrease in virus detection rates.

### 3.4. Population Genetic Analysis of Viral Coat Protein Sequences

**Figure 3:**
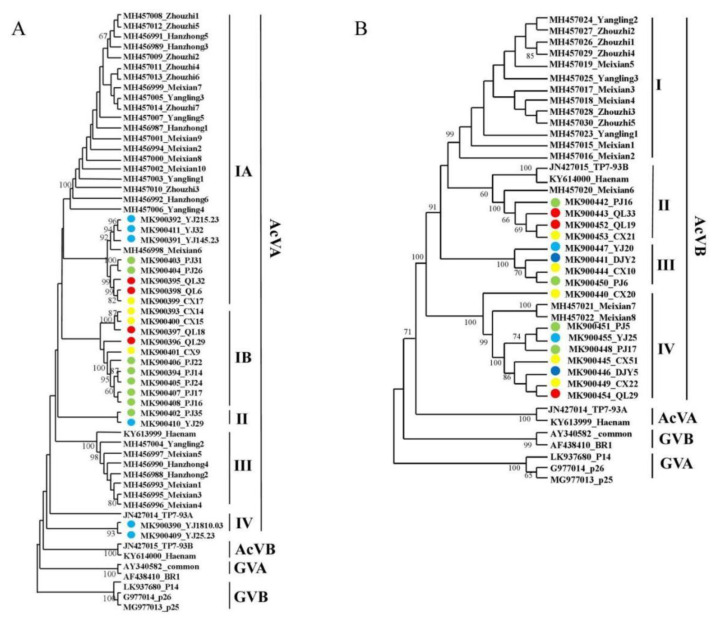
Phylogenetic analysis of AcVA (**A**) and AcVB (**B**), based on the CP genes. Note: Branches with a bootstrap value of > 60% are shown. Light blue represents the Ya’an isolate, green represents the Pujiang isolate, red represents the Qionglai isolate, yellow represents the Cangxi isolate, and dark blue represents the Dujiangyan isolate.

Phylogenetic analysis was performed on the 23 *AcVA CP* gene sequences identified in this study and 28 *AcVA CP* gene sequences identified in ‘Xuxiang’ kiwifruit. Zhao Lei et al. divided the *AcVA CP* gene sequence into two subgroups, namely subgroups I and II in ‘Xuxiang’ kiwifruit [17]. AcVA isolates from the Sichuan province were mainly distributed in subgroups I, II, and IV. The isolates collected in Qionglai were mainly distributed in subgroups IA and IB, while those from Yingjing were mainly distributed in subgroups IA, IB, and IV. The AcVA isolates in Shaanxi were mainly distributed in subgroups IA and III. The sequences of the isolates from Meixian6 and Yingjing were similar, which may be related to the similar varieties of kiwifruit planted in these two places (Figure 3A).

AcVB and AcVA are closely related in their evolution, and both belong to the genus Vitivirus. The 16 *AcVB CP* gene sequences analyzed in this study and the 16 *AcVB CP* gene sequences [17] reported in Shaanxi were analyzed for phylogenetic analysis. The AcVB isolates from the Sichuan province were mainly located in subgroups II, III, and IV; the reference sequences TP7-93A and Heanam from New Zealand and Korea, respectively, were mainly located in subgroup I; and the AcVB isolates from the Shaanxi province were mainly located in subgroup I. Among them, Meixian6, Meixian7, and Meixian8 were similar to the *AcVB CP* gene sequences of Sichuan (Figure 3B).

**Figure 4:**
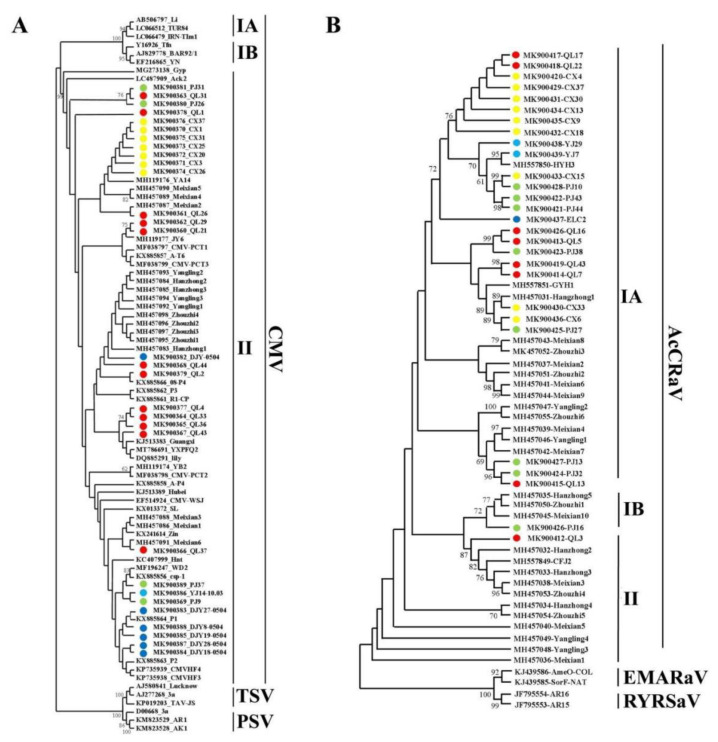
Phylogenetic analysis of CMV (**A**) and AcCRaV (**B**), based on CP genes. Note: Branches with a bootstrap value of > 60% are shown. Light blue represents the Ya’an isolate, green represents the Pujiang isolate, red represents the Qionglai isolate, yellow represents the Cangxi isolate, and dark blue represents the Dujiangyan isolate.

Cucumber mosaic virus (CMV) was divided into subgroups I and II, and subgroup I was divided into subgroup IA and subgroup IB. Phylogenetic analysis was performed on 30 *CMV CP* gene sequences measured in this study and 16 sequences of Shaanxi isolates. The results showed that all CMV isolates obtained in this study belonged to subgroup II, and some sequences from Meixian were closer to the Shaanxi isolates. However, the sequence from the Cangxi isolate was close to that from YA14 (an isolate from the Ya’an cowpea), and that from the Qionglai isolate was close to that from JY6 (an isolate from Jiangyou lettuce) (Figure 4A).

Phylogenetic analysis was performed on the 28 *AcCRaV CP* gene sequences obtained in this study and 24 sequences [17] of Shaanxi isolates (Figure 4B). The results showed that the isolates from Sichuan were mainly in subgroup IA, PJ16 was in subgroup IB, and QL3 was in subgroup II; the isolates from Shaanxi were mainly concentrated in subgroups IA, IB, and II. (Figure 4B).

### 3.5. Genetic Diversity Analysis of Kiwifruit Virus CP Genes

Among the AcVA isolates, high haplotype diversity occurred in isolates from Zhouzhi, Hanzhong, Yangling, Guangyuan, Qionglai, and other places. The Guangyuan isolate had the highest nucleotide diversity, while the Zhouzhi isolate had the lowest nucleotide diversity. AcVA may have first appeared in Guangyuan City, Sichuan province (Table S6).

Among the AcVB isolates, high haplotype diversity was found in Meixian, Yangling, Guangyuan, Ya’an, and Dujiangyan. The haplotype diversity was lowest in Qionglai. The diversity of nucleic acids was highest in Dujiangyan isolates and lowest in Yangling isolates. AcVB may have first appeared in Dujiangyan City, Sichuan province (Table S7).

Among the CMV isolates, high haplotype diversity was found in isolates from Meixian, Zhouzhi, Hanzhong, and Yangling. The haplotype diversity was lowest in the broad-element population. The diversity of nucleic acids was highest in Yangling isolates. CMV-infecting kiwifruit may have first appeared in the Yangling District, Xianyang City, Shaanxi province (Table S8).

Among the AcCRaV isolates, high haplotype diversity was found in isolates from Zhouzhi, Hanzhong, Yangling, and Ya’an. The haplotype diversity of the Guangyuan isolate was the lowest. Nucleic acid diversity was highest in the Zhouzhi isolates and lowest in the Ya’an isolates. AcCRaV may have first appeared in Zhouzhi County, Shaanxi province (Table S9).

### 3.6. Population Differentiation and Gene Flow

For the *AcVA CP* gene, the FST values of the populations between Ya’an and Pujiang, between Ya’an and Meixian, between Ya’an and Hanzhong, between Ya’an and Zhouzhi, between Ya’an and Yangling, between Pujiang and Meixian, between Pujiang and Hanzhong, between Pujiang and Zhouzhi, and between Pujiang and Yangling were greater than 0.25000, indicating that there were large genetic differences in the populations from these regions. The FST values between groups from Ya’an and Qionglai, and between groups from Ya’an and Guangyuan were between 0.15000 and 0.25000. The FST value between the populations from Pujiang and Guangyuan was less than 0.15000, indicating that there was a moderate degree of genetic differentiation in the *CP* genes of the AcVA populations from Pujiang and Guangyuan. The FST value between Pujiang and Zhouzhi was the highest, namely 0.67050. The second highest FST value was that between Ya’an and Zhou Zhi, namely 0.65041. The FST value between the populations of Pujiang and Guangyuan was the lowest at 0.12429. In general, the *CP* genes of the AcVA populations had a moderate degree of genetic differentiation among many regions. The Nm values between the populations of Ya’an and Qionglai, between those of Ya’an and Guangyuan, between those of Ya’an and Meixian, between those of Ya’an and Yangling, between those of Pujiang and Qionglai, and between of those of Pujiang and Guangyuan were all > 1.00, indicating that there was significant gene flow between the populations of these places. The results showed that the gene flow of *CP* genes in the AcVA populations was not influenced by the geographical location (Table S10).

For the *AcVB CP* gene, the FST values of the populations between Ya’an and all parts of Shaanxi, between Dujiangyan and Zhouzhi, and between Guangyuan and all parts of Shaanxi were greater than 0.25000, indicating that the populations in these regions had great genetic differentiation. The FST value between Guangyuan and Yangling was the highest. The second highest FST value was between Guangyuan and Zhouzhi. The lowest FST was observed between the populations from Pujiang and Yangling. The Nm values of the populations between Pujiang and Meixian, between Guangyuan and Meixian, and between Zhouzhi and Meixian were all above 1.00, indicating that there was significant gene flow in these areas (Table S11).

For the *CMV-CP* gene, differences were found between Pujiang and Dujiangyan; among Guangyuan, Hanzhong, Zhouzhi, Yangling, Dujiangyan, and Guangyuan; among Hanzhong, Zhouzhi, Yangling, Guangyuan, and Meixian; and among Hanzhong, Zhouzhi, and Yangling. The FST value among Meixian, Hanzhong, Zhouzhi, and Yangling was >0.25000, indicating a high genetic divergence in the virus populations among these regions. The FST values between Pujiang and Qionglai, and between Dujiangyan and Meixian were lower than 0.15000, indicating that genetic differences of the populations between these places were small. Among them, the FST value between the populations from Guangyuan and Zhouzhi was the largest (0.73236). The populations of Pujiang and Qionglai had the smallest FST value of 0.10212. The Nm values between populations from Pujiang and Qionglai, between populations from Dujiangyan and Meixian, between populations from Dujiangyan and Meixian, between populations from Meixian and Yangling, between populations from Hanzhong and Zhouzhi, and between populations from Zhouzhi and Yangling were all higher than 1.00. This result suggested that there is gene flow between these places. The largest Nm value was between the populations from Pujiang and Qionglai (4.40). There was no significant gene flow between other populations (Table S12).

For the *AcCRaV CP* gene, the FST values between populations from Ya’an and Qionglai, between populations from Ya‘an and Meixian, between populations from Ya‘an and Hanzhong, between populations from Ya’an and Zhouzhi, between populations from Ya’an and Yangling, between populations from Guangyuan and Meixian, between populations from Guangyuan and Hanzhong, between populations from Guangyuan and Zhouzhi, and between populations from Guangyuan and Yangling were all higher than 0.25000, and there were great genetic differences among the populations. The Nm values between populations from Ya’an and Pujiang, between populations from Ya’an and Guangyuan, between populations from Pujiang and Guangyuan, between populations from Pujiang and Meixian, between populations from Pujiang and Hanzhong, between populations from Pujiang and Zhouzhi, between populations from Pujiang and Yangling, between populations from Guangyuan and Meixian, and between populations from Guangyuan and Zhouzhi were all greater than 1.00, indicating gene flow between these sites. The Nm value was the largest between the populations of Pujiang and Zhouzhi (4.46). Gene flow between other regions was not obvious (Table S13).

### 3.7. Selection Pressure

The dN/dS of AcVA populations in different locations was less than 1, indicating a negative selection pressure (Table S14). The dN/dS value of the AcVB populations was less than 1 in Zhouzhi, Shaanxi province. This result suggested that *CP* genes of the AcVB populations in Zhouzhi were under negative selection pressure, whereas *CP* genes of the AcVB populations in most regions were under positive selection pressure. *CP* genes of the CMV populations in Zhouzhi and Hanzhong were under negative selection pressure. In addition to Qionglai, *CP* genes of the AcCRaV populations in other places were all under negative selection pressure. Negative selection pressure was most common in *CP* genes of the AcVA populations, followed by *CP* genes of the AcCRaV populations. (Table S14).

### 3.8. Analysis of Population Dynamics

For the *AcVA CP* genes, the scores for Tajima’s D, Fu and Li’s D, and Fu and Li’s F of the Zhouzhi, Yangling, and Guangyuan populations were all negative. The AcVA population in these regions may be in an expanding state, while the AcVA population in other regions is relatively stable (Table S15). For the *AcVB CP* genes, the scores for Tajima’s D, Fu and Li’s D, and Fu and Li’s F of the Zhouzhi population were all negative, and the Zhouzhi AcVB population might be in a dilated state. The other areas were mostly positive, and AcVB is likely to be in a relatively stable state as a whole (Table S16). For the *CMV CP* genes, the scores for Tajima’s D, Fu and Li’s D, and Fu and Li’s F in Meixian, Zhouzhi, Yangling, and Guangyuan were all negative, indicating that the CMV population in these five regions was expanding (Table S17). For the *AcCRaV CP* genes, the scores for Tajima’s D, Fu and Li’s D, and Fu and Li’s F in Meixian, Zhouzhi, Guangyuan, Qionglai, and Dujiangyan were all negative, indicating that the AcCRaV population in these regions may be in a state of expansion (Table S18).

### 3.9. Repeated Sequence Analysis of the 5′-Terminal Non-Coding Region of RNA3 in AcCRaV

**Figure 5:**
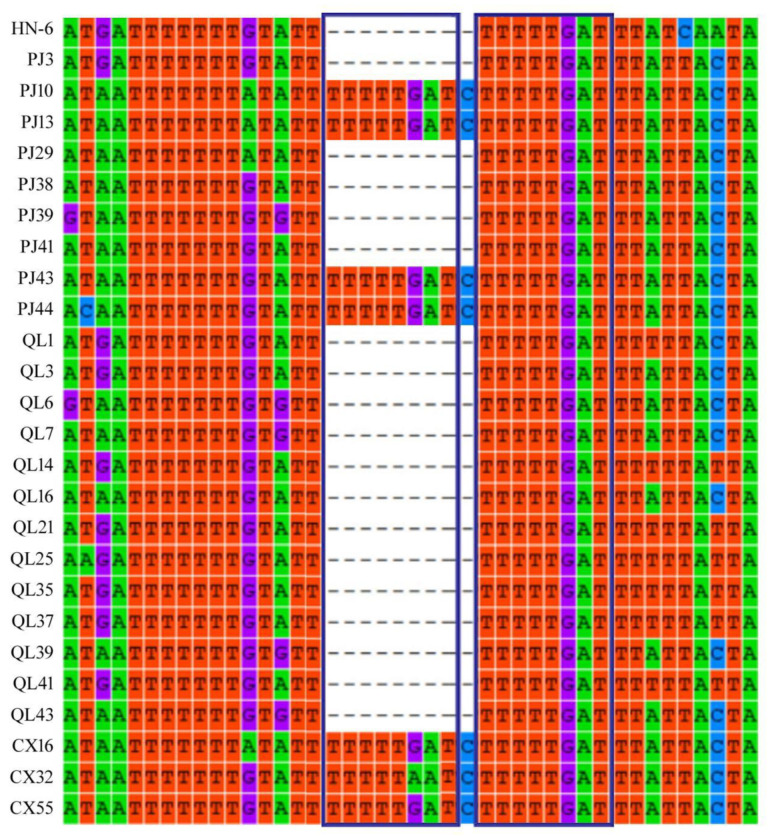
Multiple nucleotide sequence alignment of 26 AcCRaV isolates in the 5′-terminal non-coding region of RNA3.

**Figure 6:**
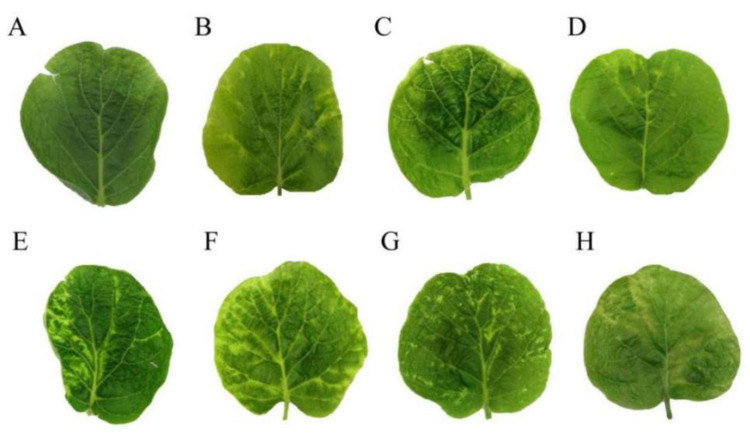
Comparison of symptoms of the 5′-terminal non-coding region insertion type and the non-insertion type of AcCRaV. Note: (**A**–**D**), 5′-terminal non-coding region insertion of AcCRaV; (**A**), PJ10; (**B**), PJ13; (**C**), PJ43; (**D**), CX5; (**E**,**F**), non-insertion AcCRaV; (**E**), PJ39; (**F**), QL3; (**G**), QL7; (**H**), QL16.

We found that the 5′-terminal non-coding region of RNA3 was highly variable (Table S19). The 461 to 471 bp fragment of the 5′-terminal non-coding region of RNA3 in the samples PJ10, PJ13, PJ43, PJ44, CX14, CX32, and CX55 had an eight-base repeat (TTTTTGAT) (Figure 5). However, sequence analysis of other isolates did not find these repeated sequences. Unique eight-nucleotide (TTTTTGAT) repeats were found in the 5′-terminal non-coding region of the AcCRaV RNA3 in a possible association with reduced disease symptoms (Figure 6).

## 4. Discussion

The main kiwifruit viruses in the Sichuan province were AcVA, AcVB, AcCRaV, and CMV. The most common infection was AcCRaV alone, followed by CMV alone. Both AcCRaV and CMV were the highest in terms of the detection rate and disease index. In addition, AcCRaV and CMV often appeared in co-infections in Sichuan. AcVA and AcVB also frequently co-infected vines. This may be due to the synergistic effect between the viruses, which promote each other to invade the host during the infection process [36]. AcVA (or AcVB) and CMV rarely co-infected kiwifruit, and may have antagonistic effects on each other.

The symptoms of AcVA and AcVB infection in kiwifruit leaves were mild, with mild shrinkage and slight chlorosis along the veins, whereas the symptoms of AcCRaV and CMV were more obvious. Kiwifruit leaves infected with CMV usually showed red at the edge of the chlorotic spots. However, the symptoms of AcCRaV were variable, including chlorotic ringspots, small chlorotic spots, and bright pulse retraction [17]. Co-infection with multiple viruses can cause the symptoms to overlap. Discrimination of the symptoms is helpful for preliminary determination of kiwifruit virus diseases in the field.

The virus detection rate was positively correlated with vine age at about 600 m. The virus detection rate is higher in orchards below 600 m than in orchards above 600 m. Low temperatures are not conducive to replication of the virus and the survival of virus-transmitting insects [37].

The phylogenetic analysis showed that AcVA isolates from the Sichuan province were mainly located in subgroups IA and IB. AcVA isolates from Shaanxi were mainly located in subgroups IA and III. AcVB isolates from the Sichuan province were mainly located in subgroups II, III, and IV. AcVB isolates from Shaanxi were mainly located in subgroup I. These differences may be caused by geographical barriers [33]. CMV isolates on kiwifruit from Sichuan were clustered in subgroup II and showed high genetic homology with CMV isolates from other hosts. This may have been caused by a lack of weed control. Weeds act as intermediate hosts and increase the spread of CMV [15]. At the same time, the transmission efficiency of aphids for CMV subgroup II was higher than that for CMV subgroup I [38]. The AcCRaV isolates from the Sichuan province were mainly located in subgroup IA and relatively clustered, while the AcCRaV isolates from the Shaanxi province contained more subgroups. This indicated that the genetic diversity of AcCRaV from Shaanxi was higher. Zhao Lei et al. ascribed Hanzhong5, Meixian10, and Zhouzhi1 to subgroup II [17]. However, in this study, as the number of sequences analyzed increased, we found that these sequences should be classified into subgroup IB. In the Sichuan province, mixed co-infection with multiple viruses may increase the difficulty of comprehensive prevention and control of kiwifruit virus diseases. Strengthening the isolation, inspection, and quarantine of introduced species in the different main production areas is a powerful measure for preventing the further spread of new strains of kiwifruit viruses.

The place with the most abundant genetic diversity of genes is presumed to be the origin of the virus [39]. This indicates that the virus may have experienced multiple gene rearrangement events. AcVA appeared earlier in Guangyuan, Sichuan. AcVB originated in Dujiangyan, Sichuan province. CMV appeared earlier in Yangling, Shaanxi. AcCRaV appeared earlier in Zhouzhi, Shaanxi. Hongyang kiwifruit is the main host of Actinidia viruses in Sichuan. Xu Xiang is the main host of the Actinidia viruses in Shaanxi. AcVA and AcVB appeared earlier in Chinese kiwifruit. CMV and AcCRaV appeared earlier in *Actinidia deliciosa*.

In terms of gene flow, most of the flow of the *AcVA CP* gene occurred between Ya’an (or Pujiang) and other places. The flow of the *AcVB CP* gene mainly occurred between Meixian County and other regions. The flow of the *CMV CP* gene mainly occurred in the Sichuan province or the Shaanxi province. CMV is a typical virus transmitted by aphids [18]. Previous studies have confirmed that there is a specific recognition between viruses and aphids [40]. The different types of aphids in different regions restrict the flow of viral genes. The flow of the *AcCRaV-CP* gene mainly occurred in Pujiang and other places. Through investigation, it was found that Pujiang is an important production base for kiwifruit germplasm resources. AcCRaV is the most prevalent virus in Sichuan. Some kiwifruit branches with viruses are used for sale. Therefore, human factors also lead to the spread of the virus.

The *AcVA CP* gene was subjected to negative selection pressure. Most *AcVB CP* and *CMV CP* genes were subjected to positive selection pressure, while most *AcCRaV CP* genes were subjected to negative selection pressure. According to the aforementioned analyses of the genetic differences and selection pressure, the *CP* genes of AcVB and CMV have been greatly influenced by the host and the natural environment [41].

From the perspective of kiwifruit viruses’ population dynamics, except for AcVB, the other three viruses showed the trend of population expansion [42]. Ongoing research into the population dynamics of kiwifruit viruses will be helpful for the prediction and control of new kiwifruit virus strains in Sichuan.

AcCRaV is widely distributed in Sichuan and was selected for full-length analysis. The 5′-terminal non-coding region plays an important role in viral transcription and replication. AcCRaV has very long non-coding regions at the 5′-terminal ends of RNA3 and RNA4, ranging from 289 bp to 645 bp. The non-coding region repeats at the 5′-terminal of AcCRaV RNA3 make the symptoms of the host milder. Viruses that are missing repetitive sequences in the 5′-terminal non-coding region aggravate the symptoms in kiwifruit leaves. Our previous study also found that recombination in the soybean mosaic virus could also alleviate the symptoms of the host [33]. More repetitive sequences make the genome of the virus longer. They often form a stem ring structure by self-pairing. This may interfere with viral gene replication and protein translation. The wide distribution and genetic diversity of AcCRaV in Sichuan need more attention and research in the future.

In conclusion, this study identified the main viruses infecting kiwifruit in Sichuan, China, and determined the incidence, distribution, and genetic diversity of these viruses. This is the first report of the molecular variability of AcVA, AcVB, AcCRaV, and CMV in Sichuan. These results provide important data for the study of kiwifruit viruses, especially the genetic evolution of the four viruses. Our study provides a basis for the development of guidelines for the prevention of the spread of kiwifruit viruses in the Sichuan province, China. At present, the effect of the viruses on kiwifruit production in China is unclear; few studies have been conducted. Consequently, there is an urgent need for further studies to determine the impact of viruses and recommend appropriate management practices.

## Figures and Tables

**Table 1:** The mean mosaic rate and disease index of viruses in the Sichuan province, China. Values with different letters in a column differ significantly (*p* < 0.05).

Virus	Mosaic Area ×10^6^ (ppi)	Total Area ×10^6^ (ppi)	Mosaic Rate	Disease Index (DAI)	Detection Rate
AcVA	0.95 ^b^	5.22 ^a^	17.07% ^a^	5.57%	9.03%
AcVB	1.15 ^b^	5.49 ^a^	24.3% ^a^	3.94%	7.10%
AcCRaV	2.12 ^ab^	4.97 ^a^	42.4% ^a^	5.47%	41.94%
CMV	2.92 ^a^	6.20 ^a^	47.86% ^a^	7.92%	9.68%
AcVA + AcVB	0.25 ^a^	5.55 ^a^	4.868% ^a^	2.58%	5.16%
AcVA + AcCRaV	1.24 ^a^	5.78 ^a^	22.85% ^a^	2.79%	7.10%
AcVB + AcCRaV	0.05 ^a^	6.36 ^a^	0.75% ^b^	1.02%	1.29%
AcVA + CMV	1.59 ^a^	5.91 ^a^	25.63% ^a^	0.90%	1.29%
AcVB + CMV	1.51 ^a^	6.00 ^a^	24.41% ^a^	1.10%	1.29%
AcCRaV + CMV	1.96 ^a^	4.91 ^a^	40.63% ^a^	3.75%	8.39%
AcVA + AcVB + AcCRaV	2.04 ^b^	5.76 ^a^	34.08% ^b^	2.94%	3.87%
AcVA + AcCRaV + CMV	1.87 ^b^	5.58 ^a^	35.49% ^b^	0.90%	1.29%
AcVB + AcCRaV + CMV	2.56 ^ab^	5.05 ^a^	51.49% ^ab^	1.49%	1.94%
AcVA + AcVB + AcCRaV + CMV	4.70 ^a^	6.49 ^a^	72.42% ^a^	0.65%	0.65%

## Data Availability

Not applicable.

## Supplementary Materials

The following supporting information can be downloaded at: https://www.mdpi.com/article/10.3390/v14112460/s1, Table S1: All the primers used in this study; Table S2: Isolates of *Actinidia* virus A (AcVA) with complete coat protein nucleotide sequences; Table S3: Isolates of *Actinidia* virus B (AcVB) with complete coat protein nucleotide sequences; Table S4: Isolates of *Actinidia* chlorotic ringspot-associated virus (AcCRaV) with complete coat protein nucleotide sequences; Table S5: Isolates of Cucumber mosaic virus (CMV) with complete coat protein nucleotide sequences; Table S6: Genetic diversity of the CP genes in the different AcVA populations; Table S7: Genetic diversity of the CP genes in the different AcVB populations; Table S8: Genetic diversity of the CP genes in the different CMV populations; Table S9: Genetic diversity of the CP genes in the different AcCRaV populations; Table S10: Population differences in the CP gene of the AcVA populations; Table S11: Population differences in the CP genes of AcVB populations; Table S12: Differences in the CP genes of CMV populations; Table S13: Differences in the CP gene of AcCRaV populations; Table S14: Selective pressure on different groups of the four viruses; Table S15: Neutrality tests for CP genes in different AcVA populations; Table S16: Neutrality tests for CP genes in different AcVB populations; Table S17: Neutrality tests for CP genes in different CMV populations; Table S18: Neutrality tests for CP genes in different AcCRaV populations; Table S19: Isolates of AcCRaV with RNA3 nucleotide sequences used for analysis. Note: Abbreviations of the region names in Table S2–S18: PJ: PuJiang; QL: QiongLai; DJY: DuJiangYan; MX: MeiXian; ZZ: ZhouZhi; YL: YangLing; GY: GuangYuan; HZ: HanZhong; YA: Ya’an; CD: ChengDu.
