# Supplementary material for: Occurrence and Molecular Variability of the Main Kiwifruit Viruses in the Sichuan Province of China
Source: Viruses. 2022 Nov 6;14(11):2460. doi: 10.3390/v14112460 (PMC9696399; doi:10.3390/v14112460)
Supplement: Supplementary file 1 [file viruses-14-02460-s001.zip › viruses-1957123-supplementary.pdf]

viruses

MDPI

Supplementary Material

Occurrence and Molecular Variability of the Main Kiwifruit Viruses in the Sichuan Province of China

Jing Shang [1,*,†], Qi Jia [1,†], Lei Zhang [1], Siqi Zhang [1], Junbo Du [1], Wenming Wang [2] and Jing Shui [3]

1 College of Agronomy, Sichuan Agricultural University, Chengdu 611130, China
2 State Key Laboratory of Crop Gene Exploration and Utilization in Southwest, Chengdu 611130, China
3 Bureau of Agriculture and Rural Affairs, Luzhou 646000, China
* Correspondence: shangjing_edu@163.com; Tel.: +028-86290872
† These authors contributed equally to this work.

Supplement Table S1. All the primers used in this study.

Name of Primers	Sequence (5′-3′)	Product Size (bp)	Use	Reference
AcVA F	CATGGCAAAGAATATCTCAAG	471	Detection of AcVA	Zhao et al.2018
AcVA R	AGATCCAACCCAGAGTTGAAA			
AcVB5F	GTTTGCGAGGAGACGTAGGGC	342	Detection of AcVB	Blouin et al. 2012 Zheng et al. 2015
AcVB5R	AGTTAAGTGCTCTYGGRGGTGTG			
AcCRaV3F	ATCCAAGAATTCCTTAACAGCA	477	Detection of AcCRaV	Zheng et al. 2017
AcCRaV3R	TGTGCAATCATGGCTTATCAGA			
ASGV-F	CCCGCTGTTGGATTTGATACACCTC	499	Detection of ASGV	James et al. 1997
ASGV-R	GGAATTTCACACGACTCCTAACCCTCC			
CTLV-AP	CCTGAATTGAAAACCTTTGCTGCC	456	Detection of CTLV	Ito et al. 2002
CTLV-AM	TAGAAAAACCACACTAACCCGGAAATC			
AMV F	GCTGGTAAACCTACTAAAC	399	Detection of AMV	Zhao et al.2018
AMV R	AAACCCGAACTTCTCATT			
CMV F	GGATGCTTCTCCRCGAGDT	870	Detection of CMV	This study
CMV R	GCTGGATGGACAACCCGTTC			
PVX F	AGTGCGCGAGGTTTACCAATC	790	Detection of PVX	Zhao et al.2018
PVX R	GTGGTTTGCCGCGAACGATTC			
CNV-F791	CCTCGCAGAAGACCTTATGC	215	Detection of CNV	Blouin et al. 2013
CNV-R1002	GCCGACTCCTCCACTCCA			
AcVA CPF	TGCCTAGCGTGTATGAAGC	644	CP gene clone for AcVA	Zhao et al.2018
AcVA CPR	CCGTGAGAAATGATGGGTC			
AcVB CPF	CTTCCCTGGTTACTTTGTG	727	CP gene clone for AcVB	This study
AcVB CPR	GTTTATTCACGCCTGTCTC			
AcCRaV CP-F	CGAGCTCAGTGGAAGAACCACAATATT	945	CP gene clone for AcCRaV	Zheng et al. 2017

AcCRaV CP-R	CGGGATCCATGCCAAAGCCTATGCAAGG			
CMV CPF	CTTTCTCATGGATGCTTCTC	885	CP gene clone for CMV	Felix and Clara 2008
CMV CPR	GCCGTAAGCTGGATGGAC			

Supplement Table S2. Isolates of *Actinidia* virus A (AcVA) with complete coat protein nucleotide sequences.

Isolate	Accession Number	Original Host	Geographical Origin	Sequence Reference
YJ1810.03	MK900390	Actinidia	Yingjing, Sichuan, China	This study
YJ145.23	MK900391	Actinidia	Yingjing, Sichuan, China	This study
YJ215.23	MK900392	Actinidia	Yingjing, Sichuan, China	This study
CX14	MK900393	Actinidia	Cangxi, Sichuan, China	This study
PJ14	MK900394	Actinidia	Pujiang, Sichuan, China	This study
QL32	MK900395	Actinidia	Qionglai, Sichuan, China	This study
QL29	MK900396	Actinidia	Qionglai, Sichuan, China	This study
QL18	MK900397	Actinidia	Qionglai, Sichuan, China	This study
QL6	MK900398	Actinidia	Qionglai, Sichuan, China	This study
CX17	MK900399	Actinidia	Cangxi, Sichuan, China	This study
CX15	MK900400	Actinidia	Cangxi, Sichuan, China	This study
CX9	MK900401	Actinidia	Cangxi, Sichuan, China	This study
PJ35	MK900402	Actinidia	Pujiang, Sichuan, China	This study
PJ31	MK900403	Actinidia	Pujiang, Sichuan, China	This study
PJ26	MK900404	Actinidia	Pujiang, Sichuan, China	This study
PJ24	MK900405	Actinidia	Pujiang, Sichuan, China	This study
PJ22	MK900406	Actinidia	Pujiang, Sichuan, China	This study
PJ17	MK900407	Actinidia	Pujiang, Sichuan, China	This study
PJ16	MK900408	Actinidia	Pujiang, Sichuan, China	This study
YJ25.23	MK900409	Actinidia	Yingjing, Sichuan, China	This study
YJ29	MK900410	Actinidia	Yingjing, Sichuan, China	This study
YJ32	MK900411	Actinidia	Yingjing, Sichuan, China	This study
p26	MG977014	grapevine	Iran	Moradi 2016
p25	MG977013	grapevine	Iran	Moradi 2016
P14	LK937680	Not stated	Greece: Athens	Unpublished
common	AY340582	Not stated	Sao Paulo State, Brazil	Unpublished
BR1	AF438410	Not stated	Southern Brazil	Nickel et al. 2001
TP7-93A	JN427014	Actinidia sp.	New Zealand	Blouin et al. 2012
Haenam	KY613999	Actinidia chinensis	South Korea	Cho 2016
TP7-93B	JN427015	Actinidia chinensis	New Zealand	Blouin et al. 2012
Haenam	KY614000	Actinidia chinensis	South Korea	Cho 2016

Supplement Table S3. Isolates of *Actinidia* virus B (AcVB) with complete coat protein nucleotide sequences.

Isolate	Accession Number	Original Host	Geographical Origin	Sequence Reference
CX20	MK900440	Actinidia	Cangxi, Sichuan, China	This study
DJY2	MK900441	Actinidia	Dujiangyan, Sichuan, China	This study
PJ16	MK900442	Actinidia	Pujiang, Sichuan, China	This study
QL33	MK900443	Actinidia	Qionglai, Sichuan, China	This study
CX10	MK900444	Actinidia	Cangxi, Sichuan, China	This study
CX51	MK900445	Actinidia	Cangxi, Sichuan, China	This study
DJY5	MK900446	Actinidia	Dujiangyan, Sichuan, China	This study
YJ20	MK900447	Actinidia	Yingjing, Sichuan, China	This study
PJ17	MK900448	Actinidia	Pujiang, Sichuan, China	This study
CX22	MK900449	Actinidia	Cangxi, Sichuan, China	This study
PJ6	MK900450	Actinidia	Pujiang, Sichuan, China	This study
PJ5	MK900451	Actinidia	Pujiang, Sichuan, China	This study
QL19	MK900452	Actinidia	Qionglai, Sichuan, China	This study
CX21	MK900453	Actinidia	Cangxi, Sichuan, China	This study
QL29	MK900454	Actinidia	Qionglai, Sichuan, China	This study
YJ25	MK900455	Actinidia	Yingjing, Sichuan, China	This study
p26	MG977014	grapevine	Iran	Moradi 2016
p25	MG977013	grapevine	Iran	Moradi 2016
P14	LK937680	Not stated	Greece: Athens	Unpublished
common	AY340582	Not stated	Sao Paulo State, Brazil	Unpublished
BR1	AF438410	Not stated	Southern Brazil	Unpublished
TP7-93A	JN427014	Actinidia sp.	New Zealand	Blouin et al. 2012
Haenam	KY613999	Actinidia chinensis	South Korea	Cho 2016
TP7-93B	JN427015	Actinidia chinensis	New Zealand	Blouin et al. 2012
Haenam	KY614000	Actinidia chinensis	South Korea	Cho 2016

Supplement Table S4. Isolates of *Actinidia* chlorotic ringspot-associated virus (AcCRaV) with complete coat protein nucleotide sequences.

Isolate	Accession Number	Original Host	Geographical Origin	Sequence Reference
QL3	MK900412	Actinidia	Qionglai, Sichuan, China	This study
QL5	MK900413	Actinidia	Qionglai, Sichuan, China	This study
QL7	MK900414	Actinidia	Qionglai, Sichuan, China	This study
QL13	MK900415	Actinidia	Qionglai, Sichuan, China	This study
QL16	MK900416	Actinidia	Qionglai, Sichuan, China	This study
QL17	MK900417	Actinidia	Qionglai, Sichuan, China	This study
QL22	MK900418	Actinidia	Qionglai, Sichuan, China	This study
QL43	MK900419	Actinidia	Qionglai, Sichuan, China	This study

CX4	MK900420	Actinidia	Cangci, Sichuan, China	This study
PJ44	MK900421	Actinidia	Pujiang, Sichuan, China	This study
PJ43	MK900422	Actinidia	Pujiang, Sichuan, China	This study
PJ38	MK900423	Actinidia	Pujiang, Sichuan, China	This study
PJ32	MK900424	Actinidia	Pujiang, Sichuan, China	This study
PJ27	MK900425	Actinidia	Pujiang, Sichuan, China	This study
PJ16	MK900426	Actinidia	Pujiang, Sichuan, China	This study
PJ13	MK900427	Actinidia	Pujiang, Sichuan, China	This study
PJ10	MK900428	Actinidia	Pujiang, Sichuan, China	This study
CX37	MK900429	Actinidia	Cangci, Sichuan, China	This study
CX33	MK900430	Actinidia	Cangci, Sichuan, China	This study
CX30	MK900431	Actinidia	Cangci, Sichuan, China	This study
CX18	MK900432	Actinidia	Cangci, Sichuan, China	This study
CX15	MK900433	Actinidia	Cangci, Sichuan, China	This study
CX13	MK900434	Actinidia	Cangci, Sichuan, China	This study
CX9	MK900435	Actinidia	Cangci, Sichuan, China	This study
CX6	MK900436	Actinidia	Cangci, Sichuan, China	This study
ELC2	MK900437	Actinidia	Dujiangyan, Sichuan, China	This study
YJ29	MK900438	Actinidia	Yingjing, Sichuan, China	This study
YJ7	MK900439	Actinidia	Yingjing, Sichuan, China	This study
HN-6	NC_038772	Actinidia	China	Unpublished
Ris54	EU885286	Sorbus aucuparia L.	Finland	Kallinen et al. 2009
Vih7	EU885277	Sorbus aucuparia L.	Finland	Kallinen et al. 2009
Kuo12	EU885281	Sorbus aucuparia L.	Finland	Kallinen et al. 2009
RYRSaV	NC_038854	Cercis canadensis	USA	Di Bello 2016

Supplement Table S5. Isolates of Cucumber mosaic virus (CMV) with complete coat protein nucleotide sequences.

Isolate	Accession Number	Original Host	Geographical Origin	Sequence Reference
QL21	MK900360	Actinidia	Qionglai, Sichuan, China	This study
QL26	MK900361	Actinidia	Qionglai, Sichuan, China	This study
QL29	MK900362	Actinidia	Qionglai, Sichuan, China	This study
QL31	MK900363	Actinidia	Qionglai, Sichuan, China	This study
QL33	MK900364	Actinidia	Qionglai, Sichuan, China	This study
QL36	MK900365	Actinidia	Qionglai, Sichuan, China	This study
QL37	MK900366	Actinidia	Qionglai, Sichuan, China	This study
QL43	MK900367	Actinidia	Qionglai, Sichuan, China	This study
QL44	MK900368	Actinidia	Qionglai, Sichuan, China	This study
PJ9	MK900369	Actinidia	Pujiang, Si chuan, China	This study
CX1	MK900370	Actinidia	Cangxi, Sichuan, China	This study
CX3	MK900371	Actinidia	Cangxi, Sichuan, China	This study
CX20	MK900372	Actinidia	Cangxi, Sichuan, China	This study

CX25	MK900373	Actinidia	Cangxi, Sichuan, China	This study
CX26	MK900374	Actinidia	Cangxi, Sichuan, China	This study
CX31	MK900375	Actinidia	Cangxi, Sichuan, China	This study
CX37	MK900376	Actinidia	Cangxi, Sichuan, China	This study
QL4	MK900377	Actinidia	Qionglai, Sichuan, China	This study
QL1	MK900378	Actinidia	Qionglai, Sichuan, China	This study
QL2	MK900379	Actinidia	Qionglai, Sichuan, China	This study
PJ26	MK900380	Actinidia	Pujiang, Sichuan, China	This study
PJ31	MK900381	Actinidia	Pujiang, Sichuan, China	This study
DJY6-0504	MK900382	Actinidia	Dujiangyan, Sichuan, China	This study
DJY27-0504	MK900383	Actinidia	Dujiangyan, Sichuan, China	This study
DJY18-0504	MK900384	Actinidia	Dujiangyan, Sichuan, China	This study
DJY19-0504	MK900385	Actinidia	Dujiangyan, Sichuan, China	This study
YJ14-10.03	MK900386	Actinidia	Yingjing, Sichuan, China	This study
DJY28-0504	MK900387	Actinidia	Dujiangyan, Sichuan, China	This study
DJY8-0504	MK900388	Actinidia	Dujiangyan, Sichuan, China	This study
PJ37	MK900389	Actinidia	Pujiang, Sichuan, China	This study
BAR92/1	AJ829778	Lycopersicon esculentum (tomato)	Spain: Barcelona	Bonnet et al. 2005
TUR84	LC066512	Rapistrum rugosum	Turkey	Ohshima et al. 2016
Li	AB506797	Lily	South Korea	Unpublished
Tfn	Y16926	tomato	Italy	Roossinck. 2002
IRN-TIm1	LC066479	Impatiens balsamina	Iran	Ohshima 2016
AR1	KM823529	Medicago sativa	Iran	Unpublished
AK1	KM823528	Medicago sativa	Iran	Unpublished
3a	D00668	Not stated	Japan	Karasawa et al. 1991
Lucknow	AJ580841	Chrysanthemum sp.	India: Lucknow	Unpublished
TAV-JS	KP019203	Chrysanthemum morifolium Ramat.	China: Wuxi Jiangsu	Zhao et al. 2015
Fny	D10538	Not stated	USA	Roossinck 2002
SL	KX013372	Lycopersicon esculentum	China	Unpublished
YN	EF216865	Not stated	China	Unpublished
CMV	DQ249298	tomato	China	Unpublished
Hnt	KC407999	tobacco	Australia	Unpublished
3a	AJ277268	Not stated		Moreno et al. 1997

Supplement Table S6. Genetic diversity of the CP genes in the different AcVA populations.

Population	Sample Size	Haplotypes	Haplotype Diversity	Nucleotide Diversity
MX	10	9	0.978	0.08043
ZZ	7	7	1.000	0.00511
HZ	6	6	1.000	0.06678
YL	5	5	1.000	0.07919

GY	4	4	1.000	0.08585
PJ	8	7	0.964	0.07119
QL	4	4	1.000	0.07383
YA	6	5	0.933	0.07036
DJY	-	-	-	-

Supplement Table S7. Genetic diversity of the CP genes in the different AcVB populations.

Population	Sample Size	Haplotypes	Haplotype Diversity	Nucleotide Diversity
MX	8	8	1.000	0.09404
ZZ	5	4	0.900	0.00637
HZ	-	-	-	-
YL	3	3	1.000	0.00447
GY	5	5	1.000	0.14238
PJ	4	3	0.833	0.12619
QL	3	2	0.667	0.11279
YA	2	2	1.000	0.16415
DJY	2	2	1.000	0.16583

Supplement Table S8. Genetic diversity of the CP genes in the different CMV populations.

Population	Sample Size	Haplotypes	Haplotype Diversity	Nucleotide Diversity
MX	6	6	1.000	0.01009
ZZ	4	4	1.000	0.00728
HZ	3	3	1.000	0.00832
YL	3	3	1.000	0.01248
GY	7	3	0.524	0.00089
PJ	4	3	0.833	0.00572
QL	12	11	0.985	0.01132
YA	-	-	-	-
DJY	6	5	0.933	0.00374

Supplement Table S9. Genetic diversity of the CP genes in the different AcCRaV populations.

Population	Sample Size	Haplotypes	Haplotype Diversity	Nucleotide Diversity
MX	10	8	0.978	0.04928
ZZ	6	6	1.000	0.05239
HZ	5	5	1.000	0.05011
YL	4	4	1.000	0.04185
GY	9	5	0.772	0.01755
PJ	8	6	0.929	0.04041
QL	8	5	0.893	0.03346
YA	2	2	1.000	0.01522
DJY	-	-	-	-

Supplement Table S10. Population differences in the CP gene of the AcVA populations.

Population	K_{ST}	P-Value[a]	Snn	P-Value	F_{ST}	Nm
YA—QL	0.14328	0.0240*	1.00000	0.0300*	0.23784	1.60
YA—PJ	0.20884	0.0060**	0.85714	0.0060**	0.33392	1.00
YA—GY	0.13024	0.0400*	0.80000	0.0240*	0.21564	1.82
YA—MX	0.14290	0.0080**	0.93750	0.0020**	0.25319	1.47
YA—HZ	0.22181	0.0040**	1.00000	0.0030**	0.34321	0.96

YA－ZZ	0.51764	0.0000***	1.00000	0.0000***	0.65041	0.27
YA－YL	0.18869	0.11741*	0.81818	0.2757ns	0.29779	1.18
PJ－QL	0.13170	0.10094*	0.83333	0.2851ns	0.23757	1.60
PJ－GY	0.06629	0.0760*	0.75000	0.0460*	0.12429	3.52
PJ－MX	0.22861	0.0000***	0.94444	0.0000***	0.36331	0.88
PJ－HZ	0.27102	0.0010**	1.00000	0.0000***	0.41475	0.71
PJ－ZZ	0.50641	0.0000***	1.00000	0.0000***	0.67050	0.25
PJ－YL	0.20884	0.0050**	0.84615	0.0040**	0.33392	1.00

Supplement Table S11. Population differences in the CP genes of AcVB populations.

**Population**	K_{ST}	**P-Value**[a]	**Snn**	**P-Value**	F_{ST}	**Nm**
YA－ZZ	0.34518	0.0580ns	0.85714	0.0580ns	0.40047	0.75
YA－YL	0.33115	0.1140ns	0.80000	0.1140ns	0.40079	0.75
PJ－MX	0.07556	0.1860ns	0.79167	0.0460*	0.13824	3.12
PJ－ZZ	0.41169	0.0060**	1.00000	0.0060**	0.53120	0.44
DJY－ZZ	0.33639	0.0410*	0.85714	0.0410*	0.39100	0.78
GY－MX	0.10028	0.0880ns	0.76923	0.0270*	0.17304	2.39
GY－ZZ	0.40074	0.0070**	1.00000	0.0060**	0.54621	0.42
GY－YL	0.34416	0.0410*	1.00000	0.0380*	0.54715	0.41
MX－ZZ	0.08566	0.1200ns	0.65385	0.1360ns	0.17999	2.28
ZZ－YL	0.34518	0.0610ns	0.85714	0.0610ns	0.40047	0.75

Supplement Table S12. Differences in the CP genes of CMV populations.

**Population**	K_{ST}	**P-Value**[a]	**Snn**	**P-Value**	F_{ST}	**Nm**
PJ－QL	0.03760	0.0370*	0.87500	0.2680ns	0.10212	4.40
PJ－DJY	0.17215	0.0070**	1.00000	0.0030*	0.27200	1.34
PJ－GY	0.52381	0.0040**	1.00000	0.0040**	0.63374	0.29
PJ－MX	0.08517	0.0300*	0.85000	0.1290ns	0.15556	2.71
PJ－HZ	0.32353	0.0240*	1.00000	0.0230*	0.44898	0.61
PJ－ZZ	0.39863	0.0390*	1.00000	0.0220*	0.53704	0.43
PJ－YL	0.27500	0.0350*	1.00000	0.0300*	0.38596	0.80
DJY－GY	0.56535	0.0010**	1.00000	0.0010**	0.69709	0.22
DJY－MX	0.08276	0.0100*	0.79167	0.0050**	0.14194	3.02
DJY－HZ	0.31685	0.0130*	0.96296	0.0020**	0.44762	0.62
DJY－ZZ	0.40952	0.0020**	1.00000	0.0010**	0.54894	0.41
DJY－YL	0.26857	0.0120*	0.77778	0.0000***	0.37600	0.83
GY－MX	0.29836	0.0010**	1.00000	0.0000***	0.42490	0.68
GY－HZ	0.58333	0.0100*	1.00000	0.0100*	0.67021	0.25
GY－ZZ	0.63907	0.0010**	1.00000	0.0010**	0.73236	0.18
GY－YL	0.50000	0.0110*	1.00000	0.0110*	0.58333	0.36
MX－HZ	0.20866	0.0130*	1.00000	0.0210*	0.35244	0.92
MX－ZZ	0.29151	0.0010**	1.00000	0.0010**	0.44333	0.63
MX－YL	0.18442	0.0070**	0.88889	0.0060**	0.30374	1.15
HZ－ZZ	0.11864	0.3530ns	0.85714	0.0780ns	0.18919	2.14
ZZ－YL	0.09859	0.059ns	0.71429	0.0260*	0.15556	2.71

Supplement Table S13. Differences in the CP gene of AcCRaV populations.

Population	K_{ST}	P-Value[a]	Snn	P-Value	F_{ST}	Nm
YA—QL	0.13201	0.0730ns	1.00000	0.176	0.34380	0.95
YA—PJ	0.07723	0.1650ns	0.80000	0.4920ns	0.23040	1.67
YA—GY	0.10344	0.095ns	0.17600	0.2250ns	0.25831	1.44
YA—MX	0.12917	0.0340*	1.00000	0.2420*	0.39824	0.76
YA—HZ	0.23971	0.0390*	0.85714	0.3720ns	0.44862	0.61
YA—ZZ	0.17087	0.0430*	1.00000	0.4830ns	0.38007	0.82
YA—YL	0.30215	0.0730ns	1.00000	0.0830ns	0.48403	0.53
PJ—GY	0.08494	0.0350*	0.68750	0.0100*	0.14824	2.87
PJ—MX	0.06361	0.0030*	0.85185	0.0000***	0.11610	3.81
PJ—HZ	0.07946	0.0250*	0.69231	0.0190*	0.14102	3.05
PJ—ZZ	0.05687	0.0330*	0.78571	0.0280*	0.10088	4.46
PJ—YL	0.10399	0.0260*	0.83333	0.0260*	0.19222	2.10
GY—MX	0.18409	0.0000***	1.00000	0.0000***	0.31145	1.11
GY—HZ	0.22158	0.0010**	0.92308	0.0010**	0.33271	1.00
GY—ZZ	0.17902	0.0020**	0.94286	0.0020**	0.27866	1.29
GY—YL	0.26050	0.0000***	0.95833	0.0000***	0.38952	0.78

Supplement Table S14. Selective pressure on different groups of the four viruses.

Region	AcVA			AcVB			CMV			AcCRaV		
	dS	dN	dN/dS	ds	dN	dN/ds	ds	dN	dN/ds	ds	dN	dN/ds
MX	0.410	0.019	0.046	0.035	0.138	3.943	0.008	0.011	1.375	0.087	0.039	0.448
ZZ	0.006	0.005	0.833	0.011	0.006	0.545	0.008	0.007	0.875	0.096	0.039	0.406
HZ	0.347	0.013	0.037	-	-	-	0.009	0.007	0.778	0.090	0.040	0.444
YL	0.412	0.015	0.036	0.005	0.005	1.000	0.005	0.016	3.200	0.050	0.036	0.720
GY	0.511	0.006	0.012	0.060	0.179	2.983	0.000	0.001	-	0.025	0.012	0.480
PJ	0.454	0.005	0.011	0.058	0.177	3.052	0.000	0.007	-	0.069	0.031	0.449
QL	0.402	0.006	0.015	0.054	0.160	2.963	0.007	0.018	2.571	0.026	0.055	2.115
CD	-	-	-	-	-	-	-	-	-	0.089	0.044	0.494

Supplement Table S15. Neutrality tests for CP genes in different AcVA populations.

Region	Tajima's D	Fu & Li's D	Fu & Li's F
MX	0.50894	-0.02373	0.12402
ZZ	-1.35933	-1.3784	-1.50632
HZ	1.06435	1.40968	1.46693
YL	-0.61584	-0.49741	-0.5667
GY	-0.25204	-0.19373	-0.25785
PJ	0.22397	0.39466	0.39605
QL	-0.043	0.15409	0.12643
YA	-	-	-
DJY	0.64902	0.79476	0.83878
total	0.08604	-0.14411	-0.06809

Supplement Table S16. Neutrality tests for CP genes in different AcVB populations.

Region	Tajima's D	Fu & Li's D	Fu & Li's F
MX	0.177	0.73052	0.67104
ZZ	-0.07339	-0.07339	-0.07686
HZ	-	-	-
YL	-	-	-
GY	-0.0526	0.24935	0.20576
PJ	0.65354	0.8505	0.87464
QL	-	-	-
YA	-	-	-
DJY	-	-	-
total	0.65404	0.72138	0.82091

Supplement Table S17. Neutrality tests for CP genes in different CMV populations.

Region	Tajima's D	Fu & Li's D	Fu & Li's F
MX	-0.35152	-0.26995	-0.32825
ZZ	-0.27814	-0.21996	-0.25542
HZ	0.42856	-0.43664	-0.49151
YL	-0.20544	-0.07251	-0.10321
GY	-0.70623	-0.14619	-0.3167
PJ	-0.26274	0.0294	0.01631
QL	-0.01201	0.29492	0.24895
YA	-	-	-
DJY	-	-	-
total	-2.30350	-3.84244	-3.91410

Supplement Table S18. Neutrality tests for CP genes in different AcCRaV populations.

Region	Tajima's D	Fu & Li's D	Fu & Li's F
MX	-0.81528	-0.76608	-0.84473
ZZ	-0.49151	-0.49151	-0.48796
HZ	-	-	-
YL	-	-	-
GY	-1.23716	-1.29591	-1.37408
PJ	1.16799	1.16799	1.12267
QL	-1.42187	-1.30929	-1.52538
YA	-	-	-
DJY	-0.49605	-0.41639	-0.46347
total	-0.28834	-0.01985	-0.14687

Supplement Table S19. Isolates of AcCRaV with RNA3 nucleotide sequences used for analysis.

Isolate	Accession Number	Original Host	Geographical Origin	Sequence Reference
QL3	MZ052112	Actinidia	Qionglai, Sichuan, China	This study
QL1	MZ052111	Actinidia	Qionglai, Sichuan, China	This study
QL6	MZ052113	Actinidia	Qionglai, Sichuan, China	This study

QL7	MZ052114	Actinidia	Qionglai, Sichuan, China	This study
QL14	MZ052115	Actinidia	Qionglai, Sichuan, China	This study
QL16	MZ052116	Actinidia	Qionglai, Sichuan, China	This study
QL21	MZ052117	Actinidia	Qionglai, Sichuan, China	This study
QL25	MZ052118	Actinidia	Qionglai, Sichuan, China	This study
QL35	MZ052119	Actinidia	Qionglai, Sichuan, China	This study
QL37	MZ052120	Actinidia	Qionglai, Sichuan, China	This study
QL39	MZ052121	Actinidia	Qionglai, Sichuan, China	This study
QL41	MZ052097	Actinidia	Qionglai, Sichuan, China	This study
QL43	MW717591	Actinidia	Qionglai, Sichuan, China	This study
PJ3	MZ052102	Actinidia	Pujiang, Sichuan, China	This study
PJ29	MZ052105	Actinidia	Pujiang, Sichuan, China	This study
PJ38	MZ052106	Actinidia	Pujiang, Sichuan, China	This study
PJ39	MZ052107	Actinidia	Pujiang, Sichuan, China	This study
PJ41	MZ052108	Actinidia	Pujiang, Sichuan, China	This study
PJ43	MW717591	Actinidia	Pujiang, Sichuan, China	This study
PJ44	MZ052110	Actinidia	Pujiang, Sichuan, China	This study
PJ13	MZ052104	Actinidia	Pujiang, Sichuan, China	This study
PJ10	MZ052103	Actinidia	Pujiang, Sichuan, China	This study
CX16	MZ052096	Actinidia	Cangci, Sichuan, China	This study
CX32	MZ052125	Actinidia	Cangci, Sichuan, China	This study
CX55	MZ052126	Actinidia	Cangci, Sichuan, China	This study
HN-6	NC_038772	Actinidia	China	Unpublished

References

Blouin, A.G., Pearson, M.N., Chavan, R.R., Woo, E.N.Y., Lebas, B.S.M., Veerakone, S., Ratti, C., Biccheri, R., MacDiarmid, R.M., Cohen, D. 2012. Viruses of Kiwifruit (*Actinidia* Species). J. Plant Pathol. 99:221-235.

Bonnet, J., Fraile, A., Sacristán, S., Malpica, J. M., García-Arenal, F. 2005. Role of recombination in the evolution of natural populations of cucumber mosaic virus, a tripartite RNA plant virus. Virology. 332:359-368.

Cho, S.Y., Kim, H., Yi, S.I. 2017. First Report of Actinidia virus A and B Infecting *Actinidia chinensis* in South Korea. Plant Dis. In press.

Di, Bello, P.L., Laney, A.G., Druciarek, T., Ho, T., Gergerich, R.C., Keller, K.E., Martin, R.R. and Tzanetakis, I. E. 2016. A novel emaravirus is associated with redbud yellow ringspot disease. Virus Res. 222:41-47.

Kallinen, A.K., Lindberg, I.L., Tugume, A.K., Valkonen, J.P.T. 2009. Detection, distribution, and genetic variability of european mountain ash ringspot-associated virus. Phytopathology. 99:344-352.

Karasawa, A. 1991. Nucleotide sequence of rna 3 of peanut stunt cucumovirus. Virology. 185.

Moradi, R., Koolivand, D., Eini, O., Hajizadeh, M. 2017. Phylogenetic analysis of two Iranian Grapevine virus A isolates using coat protein gene sequence. Iran J Genet Plant Breed. 6:48-57.

Moreno, I.M., Bernal, J.J., Garcia de Blas, B., Rodriguez-Cerezo, E. Garcia-Arenal, F. 1997. The expression level of the 3a movement protein determines differences in severity of symptoms between two strains of tomato aspermy cucumovirus. Mol. Plant Microbe Interact. 10: 171-179.

Ohshima, K., Matsumoto, K., Yasaka, R., Nishiyama, M., Takeshita, M. 2016. Temporal analysis of reassortment and molecular evolution of cucumber mosaic virus: extra clues from its segmented genome. Virology. 487:188-197.

Roossinck, M.J. 2002. Evolutionary history of Cucumber mosaic virus deduced by phylogenetic analyses. J. Virol. 76:3382-3387.

Zhao, X.T., Liu, X.X., Hong, B. 2015. Characterization of tomato aspermy virus isolated from chrysanthemum and elucidation of its complete nucleotide sequence. Acta Virol. 59:204-206.
